# Chemical Composition and Chronic Toxicity of Disc-Cultured *Antrodia cinnamomea* Fruiting Bodies

**DOI:** 10.3390/toxics10100587

**Published:** 2022-10-04

**Authors:** Shou-Chou Liu, Tung-Ying Wu, Tai-Hao Hsu, Ming-Nan Lai, Yang-Chang Wu, Lean-Teik Ng

**Affiliations:** 1 College of Biotechnology and Bioresources, Da-Yeh University, Changhua County 51591, Taiwan; 2 Department of Food Science and Nutrition, Meiho University, Pingtung 912009, Taiwan; 3 Department of Medicinal Botanicals and Foods on Health Applications, Da-Yeh University, Changhua County 51591, Taiwan; 4 Kang Jian Biotech Co., Ltd., Nantou 54245, Taiwan; 5 College of Chinese Medicine, China Medical University, Taichung 406040, Taiwan; 6 Department of Agricultural Chemistry, National Taiwan University, No.1, Sec. 4, Roosevelt Road, Taipei 10617, Taiwan

**Keywords:** *Antrodia cinnamomea*, disc culture, fruiting bodies, bioactive triterpenoids, toxicological properties

## Abstract

*Antrodia cinnamomea* (AC) is a popular fungus for use as folk medicine in health maintenance and disease prevention and treatment. Disc culture is a novel technique for producing AC fruiting bodies. This study aimed to investigate the bioactive components and toxicological properties of disc-cultured AC fruiting body powders (ACP) in rats. The HPLC technique was used to quantify the composition of bioactive triterpenoids in ACP. Toxicological properties were evaluated on male and female Sprague-Dawley rats receiving ACP orally at 200, 600, and 1000 mg/kg body weight for 90 days; the control group received only distilled water. The results show that ACP contained seven important AC index compounds, namely antcins A, B, C, K, and H, dehydrosulphurenic acid, and dehydroeburicoic acid. At the tested doses, oral ACP administration for 90 days caused no mortality, adverse effects on general health, body and organ weights, and food intake. Furthermore, no significant variations were observed in hematological and biochemical parameters among either sex of ACP-treated and control animals. An histopathological examination of vital organs showed no significant structural changes in organs, even in high-dose ACP-treated animals. This study indicated that ACP contained the major bioactive triterpenoids of AC fruiting bodies, and its no-observed-adverse-effect level (NOAEL) was 1000 mg/kg/day, about 20 times the recommended daily intake.

## 1. Introduction

*Antrodia cinnamomea* (AC; synonyms include *Antrodia camphorata* and *Taiwanofungus camphoratus*), also known as Niu-Chang-Chih, is a precious edible fungus endemic to Taiwan. It grows only in the rotting empty trunk of *Cinnamomum kanehirai*, and is traditionally used for treating chronic diseases, such as liver diseases, cancer, and hypertension [1,2]. Because of its slow-growing nature, AC collection from the wild has become increasingly difficult and unsustainable; hence, various cultivation methods have been developed. AC materials produced by different cultured techniques, such as solid-state (i.e., wood log, plastic bags, or space bags) culture, liquid culture, and Petri dish culture, have been reported to possess different chemical profiles and potency in biological activities [1,2,3,4]. Fruiting bodies of AC from wood-cultured samples have been shown to have relatively similar chemical profiles to the wild AC fruiting bodies [2,4].

Studies have shown that AC fruiting bodies and mycelia possess a broad spectrum of pharmacological properties, such as anticancer [5], anti-inflammatory [6], hepatoprotective [7], antioxidant [8], and antiobesity [9]; these beneficial health effects are mainly attributed to their secondary metabolites, such as triterpenoids, polysaccharides, ubiquinone derivatives, and maleic derivatives [1,2,4], of which the triterpenoids of ergostane and lanostane skeletons are believed to play a significant role in various physiological activities [2,10]. Among them, antcin A exhibited anti-inflammatory effects [11], antcin B and methylantcinate B induced hepatocellular carcinoma cell apoptosis [12], and antcins C and H possessed anticancer [13,14] and hepatoprotective [15] activities. Other triterpenoids, such as antcin K, dehydroeburicoic acid and eburicoic acid, possessed potent antidiabetic and antihyperlipidemic activities [16,17,18]. Antrocin was effective against lung cancer cells [19]. As ergostane-type triterpenoid and polyacetylene are only found in AC fruiting bodies, they are considered to be good index compounds for the quality control of AC materials and products [3,20].

Different artificial cultivation methods have been developed to mass-produce AC materials for repeated consumption in recent years [1,2,3,4]. Hence, evaluations of the quality and safety of these materials have become essential. Although many studies have reported on the medicinal properties of AC, there is very little information available on the chemistry and safety of AC fruiting bodies produced by a newly developed disc-cultured technique. In this study, we aimed to analyze the bioactive triterpenoid composition of the powder prepared from disc-cultured AC fruiting bodies and examine their chronic toxicity in rats.

## 2. Materials and Methods

### 2.1. Mushroom Materials

**Figure 1:**
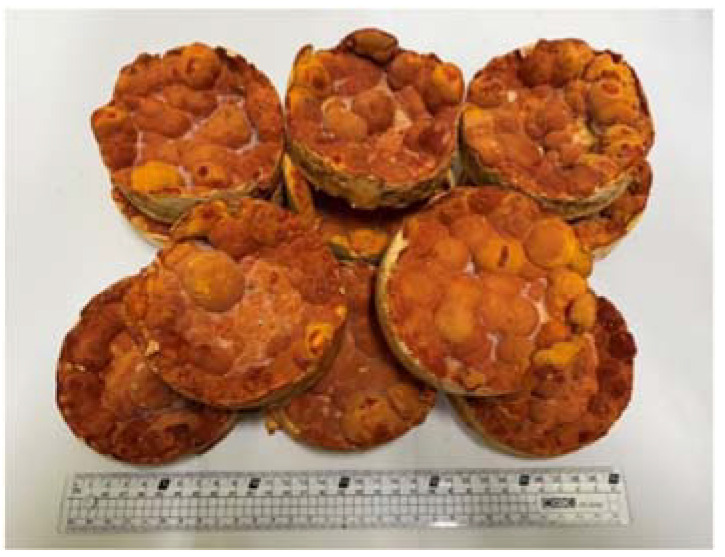
Disc-cultured *Antrodia cinnamomea* fruiting bodies obtained from 90 days of cultivation.

The powders of disc-cultured *A. cinnamomea* (AC) fruiting bodies (Figure 1) were obtained from Kang Jian Biotech Corp., Ltd. (Nantou Hsien, Taiwan). The AC culture specimen (no. KJAC-1) was deposited at Kang Jian Biotech Corp., Ltd. The AC strain was identified and confirmed by analyzing the morphological characteristics and nucleotide sequence of the internal transcribed spacers (ITS). The obtained sequences of ribosomal RNA/ITS were deposited in the GenBank at the National Center for Biotechnology Information (NCBI) database under the accession number MK764936.

In brief, the procedures of the disc-cultured method comprised the inoculation of the AC strain on the culture medium containing a fine grain powder (adlay, wheat and brown rice) plus an agar-agar powder in a disc, which was then incubated at about 25 °C for 3 months to produce the fruiting bodies.

### 2.2. Determination of Index Compounds

The analysis was performed according to the method described previously [3,20]. In brief, 200 mg of sample was taken and placed in a 15 mL microcentrifuge tube, followed by adding 8 mL of 95% alcohol (1:40, *w*/*v*), mixed well by shaking in an ultrasonic bath at room temperature for 1 h, and then centrifuged at 6000× *g* for 5 min to obtain the supernatant, which was taken and filtered through a 0.22 μm polytetrafluoroethene membrane filter, the filtrate was quantified to 1 mL with 95% alcohol and then stored at −20 °C until analysis.

The high-performance liquid chromatographic (HPLC) Agilent 1100 system (Agilent Technologies, Inc., Santa Clara, CA, USA) consisted of a G1311A Quat pump with a 20 µL fixed loop, which was connected to a Luna C18(2) column (5 µm, 4.6 × 250 mm, Phenomenex Inc., Torrance, CA, USA). The UV–visible spectroscopic detector (Agilent model G1314A) was set at 254 nm for detection. The mobile phase comprised A: H_2_O (containing 0.1% acetic acid), B: methanol, and C: acetonitrile. The flow rate of the mobile phase was 0.5 mL/min between 0 and 95 min and 1.0 mL/min between 95 and 115 min. The elution gradient at 0 min was 40% A, 30% B, and 30% C; at 5 min, 40% A, 30% B, and 30% C; at 95 min, 10% A, 10% B, and 80% C; and at 105 min, 0% A, 0% B, and 100% C.

Besides 15-acetyldehydrosulphurenic acid, the bioactive triterpenoid compounds were identified and quantified with their respective authentic standards (with purities of >99%), comprising antcin K, antcin C, antcin H, dehydrosulphurenic acid, antcin B, antcin A, and dehydroeburicoic acid. The limit of detection and the limit of quantification of these compounds were estimated to be in the range of 0.3~0.7 μg/mL and 1.0~2.0 μg/mL, respectively.

### 2.3. Animals

A total of eighty (40 females and 40 males) 5-week-old Sprague-Dawley rats were obtained from BioLASCO Taiwan Co., Ltd. (Taipei, Taiwan). All animals were maintained under a controlled environment with a 12 h light–dark cycle, a temperature range of 22 ± 2 °C, and relative humidity of 55 ± 5% throughout the experimental period. They were kept in polypropylene cages (length × width × height: 48.3 × 25.8 × 22.0 cm^3^), with two same-sex animals per cage. The rats were allowed access to standard pellet food (Oriental Yeast Co., Ltd., Tokyo, Japan) and water ad libitum. The experiments were performed after obtaining approval from the Institutional Animal Ethical Committee, with the protocol approval number KMU109113. All experimental animal procedures were conducted in accordance with the Guide for the Care and Use of Laboratory Animals (National Research Council, Washington, DC, USA, 1996).

### 2.4. Experimental Design

The chronic toxicity study was performed in compliance with the Organization for Economic Cooperation and Development (OECD) guideline 408 for testing chemicals [21]. In brief, after acclimatizing to the laboratory conditions and the gavage procedures for fifteen days, healthy animals were selected, weighed, and randomly divided into four groups, with each group contained 20 animals (10 males and 10 females). Animals of Group 1 served as controls (receiving vehicle solution only), and Groups 2–4 were administered orally (gavage) with 200 (low-dose), 600 (medium-dose), and 1000 (high-dose) mg/kg/body weight of ACP, respectively. The treatments were performed by daily oral administration of their respective doses of ACP at a fixed time every day for 90 days, while the control group received the same volume of distilled water (vehicle). The dosing volume was 10 mL/kg body weight.

During the experimental period, animals were observed twice daily for mortality, changes in general appearance or behavior, and symptoms of illness. Food intake was recorded daily, and the body weights were measured every seven days. To maintain the target dose level for all rats, the individual dose administered to the animals was adjusted weekly according to the change in body weight.

At the end of the study, all rats were fasted overnight (i.e., withdrawal of feeds only and not water), followed by anesthetizing with a dose of halothane, part of the blood samples were then collected and stored at −80 °C, while the other was collected in the nonheparinized bottle and centrifuged at 1200× *g* for 10 min to obtain the serum for hematological analysis. The animals were quickly dissected, and organs were immediately excised and freed of connective tissue and fat; they were then blotted with clean tissue paper and weighed, followed by placing in 10% neutral buffered formalin for histopathological examination. Organs such as the brain, heart, kidney, lung, adrenal gland, liver, spleen, thymus, pancreas, stomach, testes, and ovary of all animals were taken and weighed.

### 2.5. Hematological Measurements

Blood samples were subjected to the measurements of eosinophils (Eos), prothrombin time (PT), and activated partial thromboplastin time (APTT) using an automatic hematological analyzer MEK-6318K (Nihon Kohden Corp., Tokyo, Japan).

### 2.6. Biochemical Measurements

Serum samples were used to analyze aspartate aminotransferase (AST or GOT), alanine aminotransferase (ALT or GPT), alkaline phosphatase (ALP), total protein (TP), albumin (ALB), globulin (GLO), total bilirubin (TBil), gamma-glutamyl transpeptidase (GGT), blood urea nitrogen (BUN), creatinine (Cre), total cholesterols (TC) and triglycerides (TG) and glucose (GLU), as well as serum electrolytes such as sodium (Na), potassium (K), chloride (Cl), calcium (Ca), and phosphorous (P) using an automated biochemistry analyzer (Cobas Integra^®^ 400 plus, Roche, Basel, Switzerland).

On the last day of the treatment period, fresh urine was collected overnight from all animals for the measurements of specific gravity, pH, levels of leukocytes, nitrites, protein, glucose, ketones, urobilinogen, and bilirubin using an automatic urine analyzer and test strips Cybow™ (DFI Co Ltd., Gimhae, Korea).

### 2.7. Gross and Histological Examinations

Organs such as the brain, adrenal glands, heart, kidneys, lungs, liver, spleen, thymus, ovary, and testes of the test animals were excised and observed grossly. Tissue samples from each group were fixed in 10% neutral formalin, dehydrated in graded ethanol, cleaned with xylene, embedded in paraffin, cut into 3–5 μm thick sections, and then stained with hematoxylin–eosin (H&E) dye.

The description and evaluation criteria of histopathological changes were graded according to the methods described by Shackelford et al. [22]. The degree of lesions was divided into five grades: 1 = minimal (<1%); 2: slight (1–25%); 3 = moderate (26–50%); 4 = moderate/severe (51–75%); 5 = severe/high (76–100%).

### 2.8. Statistical Analysis

All data are expressed as mean ± standard deviation (SD). The microscopic features of the organs of ACP-treated rats were compared with their respective control group. The differences in pathological scores between the control group and the high-dose ACP-treated group were determined by the Student’s *t*-test. Statistical significances between means of different treatments were determined by a one-way analysis of variance (ANOVA), followed by Duncan’s multiple range test. The results were considered statistically significant if the *p*-value was less than 0.05.

## 3. Results

### 3.1. Content of Bioactive Triterpenoids

**Figure 2:**
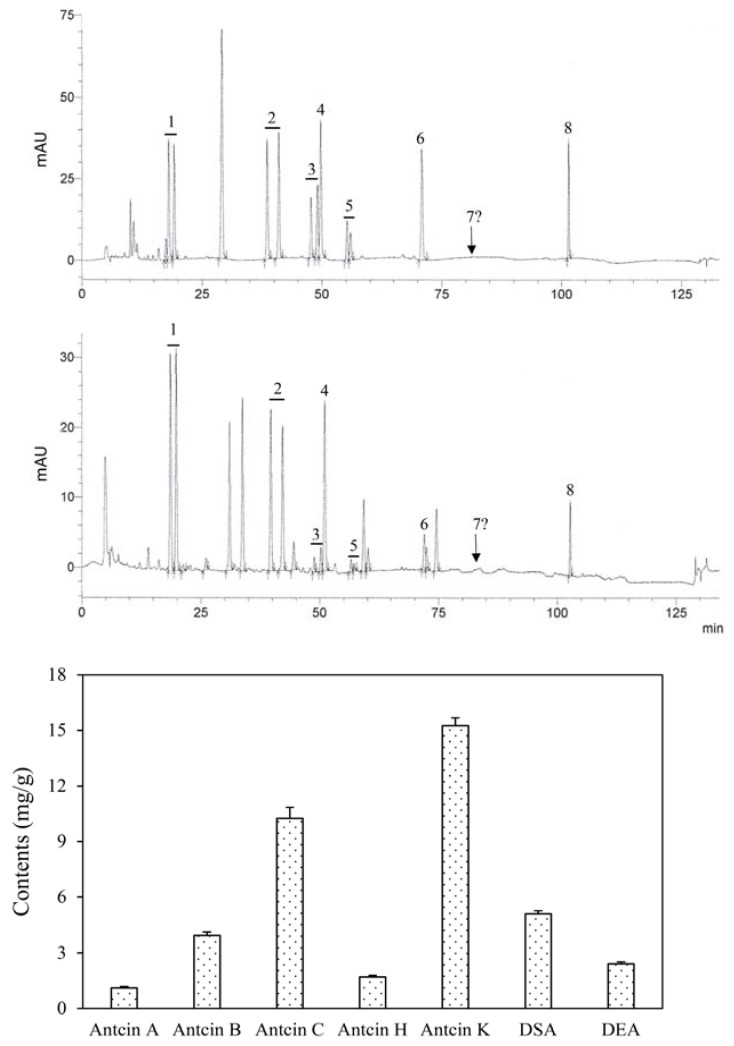
Representative chromatograms and contents of bioactive triterpenoid compounds in the powders of disc-cultured *Antrodia cinnamomea* fruiting bodies. Peaks 1 = antcin K; 2 = antcin C, 3 = antcin H, 4 = dehydrosulphurenic acid (DSA), 5 = antcin B, 6 = antcin A, 7 = 15-acetyldehydrosulphurenic acid; 8 = dehydroeburicoic acid (DEA). The underlined numbers indicate compounds that are present in both R-form and S-form, and were treated and calculated as the same compound.

The HPLC profile of ACP is shown in Figure 2, which indicates the peaks of seven well-known index compounds of AC fruiting bodies. (R,S)-antcin K, (R,S)-antcin C, (R,S)-antcin H and (R,S)-antcin B showed two peaks for the R and S configurations; for the calculation of their contents, both R-form and S-form triterpenoids were treated as the same compound. In addition to antcin A (1.12 ± 0.07 mg/g), antcin B (3.94 ± 0.18 mg/g), antcin C (10.25 ± 0.59 mg/g), antcin H (1.70 ± 0.08 mg/g) and antcin K (15.25 ± 0.43 mg/g), peaks of dehydrosulfurenic acid (5.10 ± 0.16 mg/g) and dehydroeburicoic acid (2.41 ± 0.11 mg/g) were also observed in ACP. Among the lanostane-type triterpenoids, dehydrosulfurenic acid was the most abundant compound. The content of 15-acetyldehydrosulfurenic acid, which was reported to be present in small quantity [3,20], was not quantified due to the unavailability of the reference standard.

### 3.2. Body Weight and Food Intake

Results showed that the differences in body weight changes between either sex of animals in the controls and those receiving ACP treatments were not statistically significant (Table 1). The weekly increase in body weight gains of the ACP-treated animals followed a usual trend and was insignificant compared to those of the control animals. In general, male rats were noted to have a higher body weight and food intake than female rats. During the 90 days of the study, the increases in body weight and food consumption were considered of no clinical significance and were not dose-responsive.

### 3.3. Mortality, Clinical Symptoms, and Organ Weights

Results showed that a 90-day repeated oral intake of ACP up to 1000 mg/kg did not cause any abnormal symptoms in rats of either sex. During the test period, no clinical signs such as hair loss, trauma, abnormal breathing, and abnormal eyes were noted in the control group and low-, medium- and high-dose ACP-treated animals of both sexes. Furthermore, the administration of ACP at all test doses caused no animal mortality.

Table 2 shows that the absolute organ weights of ACP-treated rats were within the normal ranges and were not significantly different from the control group of the same sex. ACP at tested doses did not change the relative weight of organs (i.e., brain, adrenal glands, spleen, heart, lung, liver, spleen, kidneys, testes, ovary, and thymus) of test animals in relation to the control group.

### 3.4. Hematological Parameters

The results on hematological parameters revealed no significant differences between the control and animals receiving different doses of ACP in the same-sex groups (Table 3). There were also no treatment-related or statistically significant effects observed in any blood chemistry parameters of animals.

### 3.5. Biochemical Parameters

Compared to the control animals, the results of the serum biochemical analysis revealed no significant alteration in the tested parameters of ACP-treated rats of either sex (Table 4). The levels of sALT, sAST, and sALP between animals of different treatments in the same sex group were not statistically significant. In addition, no significant variations were observed in the urine pH, specific gravity, glucose, protein, bilirubin, urobilinogen, and ketone in both sexes of animals when compared to the control groups (Tables S1 and S2). No blood and WBC were found present in the urine of ACP-treated rats. These results indicate that the liver and kidney function parameters were not significantly affected by a 90-day repeated ACP treatment.

### 3.6. Histopathological Observations

**Figure 3:**
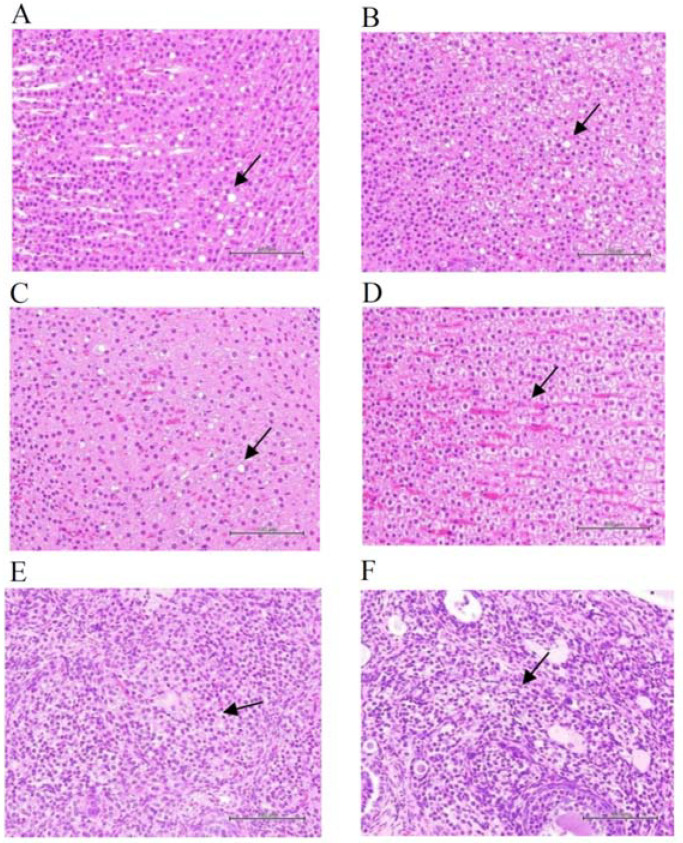
Histopathological changes of the adrenal glands of male and female rats and ovaries of female rats receiving high-dose ACP for 90 days. Arrows indicate adrenal glands of zona fasciculate having multifocal cholesterol hypertrophy and lipidosis: in the male rats of (**A**) the control (animal #111) and (**B**) high-dose ACP-treated (animal #137) groups and the female rats of (**C**) the control (animal #169) and (**D**) high-dose ACP-treated (animal #186) groups. Arrows indicate multifocal cholesterol hypertrophy of interstitial glands in ovaries of (**E**) the control (animal #169) and (**F**) high-dose ACP-treated (animal #199) female rats. In the male rats, the mean scores of cholesterol hypertrophy and lipidosis were 2.0 ± 0.0 and 0.6 ± 0.9 in the control group, and 2.3 ± 0.6 and 0.8 ± 1.0 in the high-dose ACP-treated group, respectively. In the female rats, the mean scores of cholesterol hypertrophy and lipidosis were 1.4 ± 0.9 and 0.2 ± 0.6 in the control group and 2.1 ± 0.8 and 0 in the high-dose ACP-treated group, respectively. The mean scores of multifocal cholesterol hypertrophy of interstitial glands in the ovary were 0.2 ± 0.6 and 0.5 ± 1.0 in the control and high-dose ACP-treated groups. The scores in histopathological changes between the control and high-dose ACP-treated animals were not statistically different as analyzed by the Student’s *t*-test. H&E stain; 400×.

Histopathological examination of vital organs of ACP-treated animals showed a typical structure and the absence of gross pathological lesions (Table S3). Incidental histological abnormalities were noted in certain male and female animals of the control and high-dose ACP-treated animals; for example, the adrenal glands showed multifocal, slight to moderate cholesterol hypertrophy, and lipidosis of zona fasciculate cells in control (Figure 3A,C) and high-dose ACP-treated (Figure 3B,D) animals of both sexes, and interstitial cell hypertrophy in ovaries of the control (Figure 3E) and high-dose ACP-treated (Figure 3F) female rats. The incidences and severity scores of the cholesterol hypertrophy and lipidosis of zona fasciculate cells and interstitial cell hypertrophy in ovaries were not different between the control and high-dose ACP-treated animals (Figure 3); these observations suggest that the histological abnormalities were a coincidence, and there was no positive correlation between the test substance and the occurrence of lesions.

Regardless of the sex of the animals and doses of the ACP treatment, there were no apparent differences in the histological appearance of the brain, heart, kidney, liver, lung, spleen, thymus, and testes between the control and high-dose ACP-treated animals (Figures S1 and S2). These results indicate that repeated oral ACP treatments at tested doses for 90 days did not cause adverse effects to the animal organs of either sex.

## 4. Discussion

Mushrooms and mushroom-derived products have long been used in folk medicine to prevent and treat diseases in Asian countries. Antcins are the typical triterpenoid compounds of AC fruiting bodies. Among them, antcins B, C, H, and K possess anti-inflammatory, anticancer, and hepatoprotective activities [2,3,23]. The concentration of triterpenoids in AC fruiting bodies was approximately 2.3 times higher than in mycelia [24], and ergostane-type triterpenoids were only found in AC fruiting bodies [3,20]. This study demonstrated that bioactive triterpenoid compounds present in ACP were in good agreement with those previously identified in wild AC fruiting bodies, suggesting that the disc-cultured technique could produce fruiting bodies of AC with good quality.

Changes in general behavior and body weight of test animals are considered good indicators of early signs of toxicity caused by drugs and chemicals [25]. In this study, the administration of ACP up to 1000 mg/kg body weight for 90 days caused no mortality and signs of toxicity. ACP-treated rats gained weight with age at a rate not significantly different from the mean body weight of the control animals. There was also no significant reduction in food intake in either sex of animals, suggesting that ACP did not alter the normal metabolism of carbohydrates, protein, and fats in the rats. It may also signify that the nutritional status (weight gain and appetite stability) was not adversely affected by ACP consumption.

Organ weight changes have been considered a sensitive indicator of chemically induced organ toxicity [26]. This study showed that ACP at tested doses caused no significant effect on the organs of animals of either sex. Compared to the control group, the organ relative weights also showed no significant alterations related to the treatments; these observations suggest that repeated oral administration of ACP did not negatively affect the expected growth of rats and relative organ weights.

A hematological evaluation is commonly used to determine the extent of the harmful effect of a test substance on the blood. The present study revealed no significant impacts of ACP on RBC, MCV, and Hb values of the treated rats when compared to the control animals; these observations suggest that the erythropoiesis, morphology, or osmotic fragility of RBC were not affected by the long-term ACP consumption in both sexes. The insignificant changes in neutrophils, lymphocytes, and monocytes witnessed in all tested doses also further justified the safety potential of ACP.

Liver inflammation or liver cell damage is characterized by an abnormal increase in the levels of serum enzymes such as AST, ALT, and ALP [27], sensitive biomarkers of hepatocellular function. Liver or heart damage was shown to increase sAST and sALT [28,29]. ACP at the tested doses, even as high as 1000 mg/kg body weight, caused no significant effects on the hepatic function indices. The control and ACP-treated rats exhibited comparable sAST, sALT, and sALP levels; these results suggest that ACP caused no deleterious effect on the liver and heart.

The liver mainly regulates serum cholesterols and proteins, and any changes in serum concentrations of these parameters suggest some alterations in liver functions [30,31]. Bilirubin is a breakdown product of old red blood cells and Hb, and the increase in its serum level is known to associate with primary biliary cirrhosis and cholestatic liver disease [32]. An abnormal reduction in total protein, albumin, and globulin levels indicates impaired hepatocellular function. In this study, both sexes of ACP-treated animals showed no significant variations in serum total protein, albumin, and bilirubin levels when compared to the control group. These results were consistent with the findings on hematological parameters and liver marker enzymes, which indicated that ACP had no adverse effects on the erythropoietic system and liver.

Alterations in parameters such as blood urea, creatinine, uric acid, and serum electrolytes from normal ranges may reflect renal problems and nephron dysfunction [25,33,34]. Compared to the control animals, ACP at tested doses caused no significant change in creatinine, urea, and uric acid levels. These observations indicate that ACP did not induce alterations in renal function or cause kidney damage; hence it is safe to consider that ACP did not induce harmful effects on kidneys, which was further supported by the results of the serum electrolytes (i.e., Na, K, and Cl) analysis, showing no significant changes in ACP-treated animals as compared to those of the control group.

Although histological observations showed some abnormalities in the fascicular epithelial cells of the adrenal cortex, ovary, and heart of certain animals (i.e., animal #111, animal #137, animal #169, animal #186, and animal #199), there was no positive correlation between the degree and incidence of the disease in the control group and the high-dose ACP-treated animals, suggesting that it was a nonspecific disease and had nothing to do with the test substance. The histopathological results showed that ACP did not cause apparent changes in color, shape, size, and texture of the brain, liver, heart, lungs, spleen, testes, ovary, thymus, and kidneys of animals. These results further support the findings of hematological and biochemical measurements, indicating that ACP was not harmful to vital organs.

AC materials (including fruiting bodies, mycelia, and their mixtures) produced by different methods contained various concentrations and profiles of bioactive compounds [1,2,3,4], which may contribute to the differences in pharmacological activities and potencies, as well as safety margins. Previous toxicological studies showed that the NOAEL of AC from submerged culture was greater than 3000 mg/kg BW/day in Sprague-Dawley rats [35]. AC mycelia extracts as high as 1666.67 mg/kg/day orally administered to male and female BALB/c mice caused no significant differences in body weight and hematological and biochemical parameters among the control and treatment groups [36]. Furthermore, a treatment with LDAC (700–2800 mg/kg/day), an AC powder consisting of 5% extract of fruiting bodies from cut-log cultivation, 94% mycelium from a solid-state cultivation, and 1% magnesium stearate caused no observable segment II reproductive and developmental toxic effects in pregnant SD rats and their fetuses [37]. Consistently, the present study also demonstrated that the disc-cultured AC fruiting bodies possessed low oral toxicity.

## 5. Conclusions

This study demonstrated that the disc-cultured technique could be successfully used to produce AC fruiting bodies, which (ACP) contain major bioactive triterpenoids similar to the wild AC. The long-term oral administration of ACP at the tested doses caused no clinical symptoms, mortality, changes in body weight and organ weight, and abnormalities of hematological and biochemical parameters in rats of both sexes. The histopathological examination results also showed that the continuous feeding of ACP at a high dose (1000 mg/kg body weight) for 90 days did not cause toxicity to organs such as adrenal glands, ovaries, and other important organs of female and male rats. These results indicate that ACP has a high margin of safety in rats with NOAEL of up to 1000 mg/kg body weight per day and can be regarded as safe and of low toxicity.

## Figures and Tables

**Table 1:** Changes in body weight and food intake of male and female rats treated with or without ACP for 90 days.

		ACP (mg/kg)		
	Control	200	600	1000
Male rats				
Initial body weight (g)	183.9 ± 9.1	185.8 ± 9.7	185.6 ± 6.8	187.4 ± 4.5
Final body weight (g)	511.0 ± 50.4	504.0 ± 23.2	510.0 ± 39.7	515.0 ± 35.0
Total food intake (g)	375.3 ± 16.9	374.1 ± 38.7	374.7 ± 33.9	373.1 ± 18.1
Female rats				
Initial body weight (g)	160.9 ± 4.4	160.4 ± 3.7	162.3 ± 4.6	161.1 ± 4.0
Final body weight (g)	285.0 ± 8.5	300.0 ± 18.9	306.0 ± 19.6	301.0 ± 23.3
Total food intake (g)	256.5 ± 20.0	258.8 ± 11.4	259.4 ± 25.1	260.6 ± 17.5

Values are mean ± SD, n = 10. Control: received vehicle solution only.

**Table 2:** Absolute organ weights of male and female rats treated with or without ACP for 90 days.

		ACP (mg/kg)		
	Control	200	600	1000
Male rats (Organ weights, g)				
Brain	2.24 ± 0.08	2.21 ± 0.08	2.26 ± 0.10	2.25 ± 0.09
Adrenal glands	0.06 ± 0.01	0.06 ± 0.1	0.06 ± 0.01	0.06 ± 0.01
Heart	1.70 ± 0.17	1.64 ± 0.09	1.65 ± 0.09	1.61 ± 0.13
Kidneys	3.23 ± 0.35	3.07 ± 0.17	3.19 ± 0.32	3.16 ± 0.35
Liver	13.66 ± 1.93	13.01 ± 1.38	13.66 ± 1.70	13.24 ± 1.60
Spleen	0.69 ± 0.09	0.74 ± 0.10	0.70 ± 0.07	0.69 ± 0.08
Testes	3.53 ± 0.29	3.72 ± 0.38	3.47 ± 0.29	3.67 ± 0.33
Thymus	0.29 ± 0.06	0.28 ± 0.06	0.30 ± 0.04	0.34 ± 0.08
Lung	1.71 ± 0.24	1.74 ± 0.18	1.89 ± 0.17	1.76 ± 0.12
Female rats (Organ weights, g)				
Brain	2.07 ± 0.15	2.02 ± 0.15	2.09 ± 0.12	2.06 ± 0.13
Adrenal glands	0.07 ± 0.01	0.07 ± 0.01	0.07 ± 0.02	0.07 ± 0.02
Heart	0.97 ± 0.10	1.02 ± 0.11	1.11 ± 0.08	1.02 ± 0.07
Kidneys	1.82 ± 0.14	1.94 ± 0.24	1.89 ± 0.18	1.89 ± 0.21
Liver	8.93 ± 0.88	8.63 ± 0.63	9.03 ± 0.79	8.42 ± 0.13
Spleen	0.47 ± 0.04	0.47 ± 0.03	0.49 ± 0.05	0.48 ± 0.06
Ovaries	0.10 ± 0.02	0.13 ± 0.02	0.12 ± 0.03	0.11 ± 0.02
Thymus	0.34 ± 0.10	0.37 ± 0.14	0.34 ± 0.11	0.38 ± 0.14
Lung	1.26 ± 0.16	1.24 ± 0.13	1.30 ± 0.09	1.35 ± 0.09

Values are mean ± SD, n = 10. Control: Received vehicle solution only.

**Table 3:** Hematological parameters of male and female rats treated with or without ACP for 90 days.

		ACP (mg/kg)		
	Control	200	600	1000
Male rats				
WBC (10^3^/µL)	12.01 ± 2.69	11.81 ± 3.75	12.50 ± 1.80	12.91 ± 2.69
RBC (10^6^/µL)	8.67 ± 0.34	8.71 ± 0.32	8.61 ± 0.23	8.52 ± 0.32
RDW (%)	15.75 ± 0.21	15.89 ± 0.17	15.69 ± 0.26	15.86 ± 0.22
Hb (g/dL)	16.28 ± 0.29	16.53 ± 0.29	16.40 ± 0.25	16.80 ± 0.51
Hct (%)	43.77 ± 3.23	45.50 ± 1.71	44.98 ± 1.97	44.77 ± 2.70
MCV (fL)	53.44 ± 1.81	53.51 ± 1.45	52.45 ± 1.94	54.14 ± 1.17
MCH (pg)	20.57 ± 0.51	20.87 ± 0.39	20.52 ± 0.52	20.07 ± 0.49
MCHC (g/dL)	33.57 ± 0.39	32.90 ± 0.41	33.11 ± 0.27	33.05 ± 0.36
Plt (10^3^/µL)	873.7 ± 58.0	861.5 ± 70.6	873.9 ± 88.0	877.3 ± 73.0
MPV (fL)	7.65 ± 0.52	7.64 ± 0.40	7.82 ± 0.46	7.83 ± 0.45
Lym (%)	74.42 ± 3.81	75.93 ± 4.56	79.23 ± 3.48	74.68 ± 3.66
Bas (%)	0.14 ± 0.10	0.30 ± 0.16	0.41 ± 0.23	0.35 ± 0.12
Mono (%)	0.43 ± 0.11	0.44 ± 0.08	0.45 ± 0.11	0.58 ± 0.12
Eos (%)	0.13 ± 0.03	0.15 ± 0.04	0.11 ± 0.09	0.15 ± 0.04
PT (sec)	10.88 ± 0.65	10.70 ± 0.75	10.80 ± 0.70	11.42 ± 1.25
APTT (sec)	23.21 ± 0.14	23.48 ± 0.33	23.32 ± 0.38	23.26 ± 0.34
Female rats				
WBC (10^3^/µL)	11.15 ± 3.27	10.90 ± 1.36	10.97 ± 1.16	11.09 ± 15.96
RBC (10^6^/µL)	8.06 ± 1.32	7.81 ± 0.52	7.99 ± 0.57	7.86 ± 0.48
RDW (%)	15.72 ± 0.23	15.86 ± 0.15	15.97 ± 0.24	15.90 ± 0.34
Hb (g/dl)	15.57 ± 0.69	14.36 ± 0.98	14.71 ± 0.95	14.67 ± 0.84
Hct (%)	45.05 ± 7.01	42.71 ± 2.66	44.02 ± 3.08	44.66 ± 2.71
MCV (fl)	56.00 ± 1.04	54.73 ± 1.63	55.11 ± 0.83	56.84 ± 1.24
MCH (pg)	20.05 ± 0.71	19.62 ± 0.55	20.53 ± 1.08	21.27 ± 0.95
MCHC (g/dL)	33.32 ± 0.50	33.62 ± 0.59	33.42 ± 0.59	32.85 ± 0.46
Plt (10^3^/µL)	840.9 ± 64.8	838.6 ± 55.8	889.1 ± 81.8	857.6 ± 76.9
MPV (fL)	7.47 ± 2.35	7.80 ± 0.31	7.86 ± 0.39	7.72 ± 0.43
Lym (%)	80.89 ± 3.69	74.26 ± 6.47	79.67 ± 5.52	75.72 ± 8.24
Bas (%)	0.35 ± 0.14	0.34 ± 0.20	0.45 ± 0.13	0.49 ± 0.07
Mono (%)	0.52 ± 0.14	0.52 ± 0.16	0.51 ± 0.17	0.62 ± 0.15
Eos (%)	0.16 ± 0.09	0.14 ± 0.06	0.14 ± 0.02	0.20 ± 0.23
PT (sec)	10.84 ± 0.50	10.83 ± 0.75	10.53 ± 0.35	10.84 ± 0.86
APTT (sec)	23.59 ± 0.23	23.58 ± 0.23	25.22 ± 0.90	24.24 ± 1.18

Values are mean ± SD, n = 10. Control: received vehicle solution only; WBC: white blood cell; RBC: red blood cell; RDW: red cell distribution width; Hb: hemoglobin; Hct: hematocrit; MCV: mean corpuscular volume; MCH: mean corpuscular hemoglobin; MCHC: mean corpuscular hemoglobin concentration; Plt: platelet count; MPV: mean platelet volume; Lym: lymphocytes; Bas: basophils; Mono: monocytes; Eos: eosinophils; PT: prothrombin time; APTT: activated partial thromboplastin time.

**Table 4:** Biochemical parameters of male and female rats treated with or without ACP for 90 days.

		ACP (mg/kg)		
	Control	200	600	1000
Male rats				
AST (U/L)	74.22 ± 5.12	74.50 ± 3.24	73.20 ± 6.78	72.50 ± 4.55
ALT (U/L)	30.80 ± 3.74	30.90 ± 4.70	29.30 ± 3.77	31.20 ± 4.69
ALP (U/L)	83.50 ± 6.75	86.10 ± 745	82.10 ± 9.62	82.60 ± 13.7
TP (g/dL)	6.30 ± 0.70	6.72 ± 0.39	7.03 ± 0.48	6.93 ± 0.30
ALB (g/dL)	3.40 ± 0.34	3.53 ± 0.19	3.64 ± 0.20	3.64 ± 0.13
GLO (g/dL)	2.09 ± 0.13	2.05 ± 0.19	1.97 ± 0.16	2.01 ± 0.12
TBIL (mg/dL)	0.05 ± 0.01	0.05 ± 0.01	0.04 ± 0.01	0.04 ± 0.01
GGT (U/L)	2.40 ± 1.26	2.30 ± 0.67	2.20 ± 0.63	2.60 ± 0.97
BUN (mg/dL)	16.15 ± 0.91	15.38 ± 0.97	15.11 ± 0.70	15.60 ± 0.35
Cre (mg/dL)	0.55 ± 0.11	0.58 ± 0.12	0.56 ± 0.07	0.53 ± 0.07
TC (mg/dL)	80.60 ± 5.44	78.90 ± 3.73	79.90 ± 4.43	80.10 ± 5.04
TG (mg/dL)	38.60 ± 4.03	38.80 ± 4.52	39.10 ± 8.29	40.00 ± 6.27
GLU (mg/dL)	108.6 ± 11.8	111.1 ± 9.62	124.2 ± 15.6	133.5 ± 9.01
Na (mmol/L)	147.2 ± 2.66	147.2 ± 1.32	147.3 ± 1.70	147.2 ± 1.55
K (mmol/L)	5.02 ± 0.72	5.10 ± 0.49	5.02 ± 0.36	5.21 ± 0.38
Cl (mmol/L)	102.1 ± 1.85	100.7 ± 1.57	101.6 ± 1.07	102.5 ± 0.97
Ca (mg/dL)	9.35 ± 0.41	9.81 ± 0.26	9.50 ± 0.49	9.50 ± 0.22
P (mg/dL)	7.89 ± 0.60	7.80 ± 1.33	7.79 ± 0.45	7.83 ± 0.43
Female rats				
AST (U/L)	72.90 ± 7.13	72.80 ± 4.39	74.10 ± 4.72	72.40 ± 5.10
ALT (U/L)	31.40 ± 3.69	29.40 ± 4.03	31.60 ± 4.35	30.10 ± 4.01
ALP (U/L)	76.90 ± 12.68	76.40 ± 10.74	76.10 ± 8.39	75.00 ± 11.30
TP (g/dL)	6.70 ± 0.65	7.17 ± 0.46	7.32 ± 0.40	6.96 ± 0.50
ALB (g/dL)	3.77 ± 0.34	3.89 ± 0.31	4.11 ± 0.24	3.91 ± 0.32
GLO (g/dL)	2.00 ± 0.08	2.01 ± 0.11	2.02 ± 0.13	1.98 ± 0.20
TBIL (mg/dL)	0.03 ± 0.01	0.02 ± 0.01	0.02 ± 0.01	0.03 ± 0.01
GGT (U/L)	2.30 ± 0.67	2.50 ± 0.97	2.15 ± 0.57	2.40 ± 1.07
BUN (mg/dL)	15.43 ± 0.40	15.19 ± 0.72	14.19 ± 0.46	14.38 ± 0.35
Cre (mg/dL)	0.50 ± 0.07	0.48 ± 0.14	0.49 ± 0.08	0.48 ± 0.06
TC (mg/dL)	79.20 ± 4.24	80.40 ± 6.35	79.70 ± 4.45	80.00 ± 4.83
TG (mg/dL)	37.70 ± 3.31	38.60 ± 3.84	39.00 ± 2.11	37.70 ± 4.11
GLU (mg/dL)	113.5 ± 15.7	119.0 ± 14.0	125.2 ± 15.5	131.5 ± 6.10
Na (mmol/L)	143.9 ± 1.10	145.4 ± 1.07	144.7 ± 1.06	145.6 ± 0.70
K (mmol/L)	5.19 ± 0.53	4.82 ± 0.45	4.43 ± 0.27	4.95 ± 0.41
Cl (mmol/L)	102.7 ± 1.25	102.4 ± 1.90	102.0 ± 1.33	103.3 ± 1.95
Ca (mg/dL)	9.75 ± 0.40	10.07 ± 0.30	10.08 ± 0.34	9.93 ± 0.36
P (mg/dL)	7.60 ± 0.73	7.90 ± 0.96	7.87 ± 0.47	7.98 ± 0.43

Values are ± SD, n = 10. Control: received vehicle solution only; AST: aspartate aminotransferase; ALT: alanine aminotransferase; ALP: alkaline phosphatase; TP: total protein; ALB: albumin; GLO: globulin; TBIL: total bilirubin; GGT: gamma-glutamyl transpeptidase; BUN: blood urea nitrogen; Cre: creatinine; TC: total cholesterol; TG: total triglycerides; GLU: glucose.

## Data Availability

The data used to support this research’s findings are included in the article.

## Supplementary Materials

The following supporting information can be downloaded at: https://www.mdpi.com/article/10.3390/toxics10100587/s1, Figure S1. Histopathological appearances of the organs in control rats, Figure S2. Histopathological appearances of the organs in high-dose ACP-treated rats, Table S1. Urinalysis findings of male rats receiving different doses of ACP for 90 days, Table S2. Urinalysis findings of female rats receiving different doses of ACP for 90 days, Table S3. Summary of pathological incidence in organs of rats treated with or without ACP for 90 days.
