# Supplementary material for: Chemical Composition and Chronic Toxicity of Disc-Cultured Antrodia cinnamomea Fruiting Bodies
Source: Toxics. 2022 Oct 4;10(10):587. doi: 10.3390/toxics10100587 (PMC9610047; doi:10.3390/toxics10100587)
Supplement: Supplementary file 1 [file toxics-10-00587-s001.zip › toxics-1942250-supplementary.pdf]

Supplementary Materials

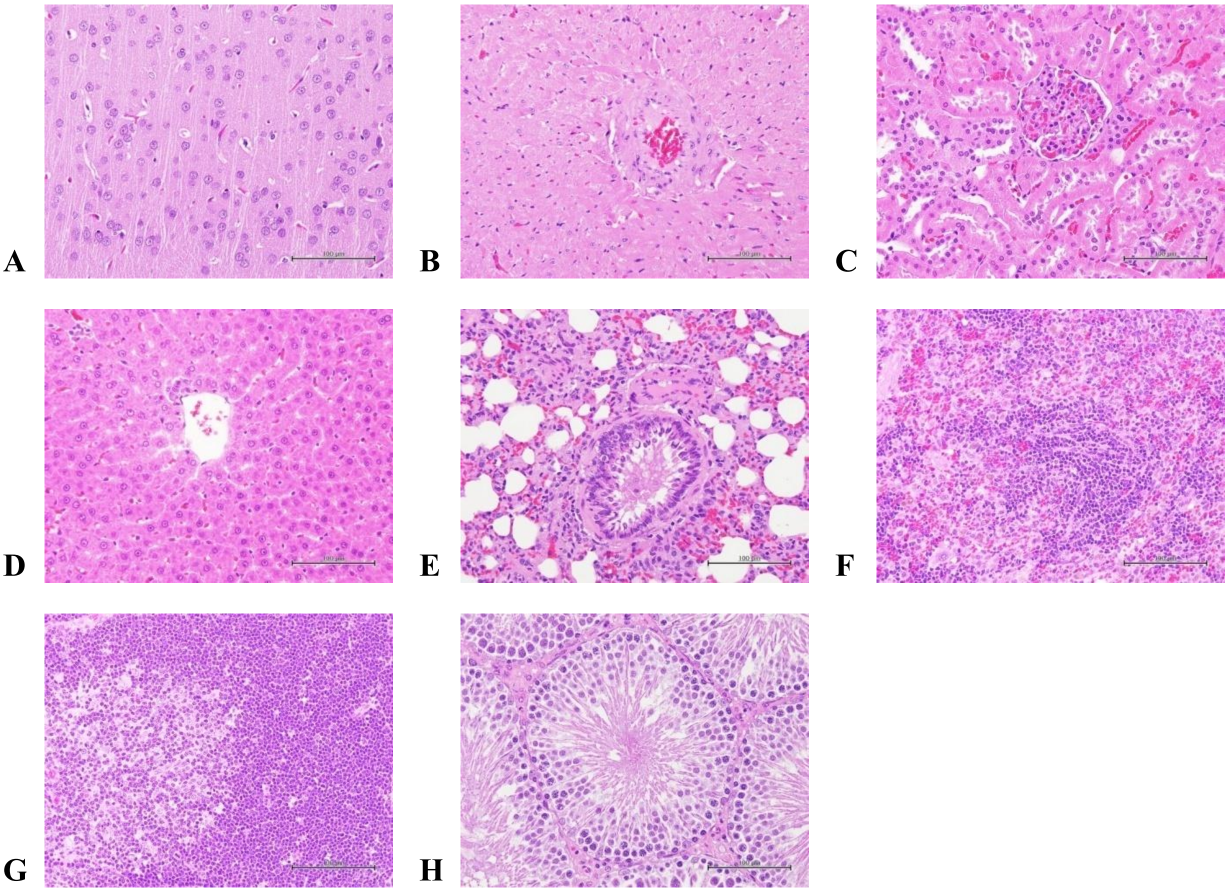

Figure S1. Histopathological appearances of the organs in control rats. No significant lesions of brain (A), heart (B), kidney (C), liver (D), lung (E), spleen (F), thymus (G), and testis (H) (animal #111). H&E stain, 400x.

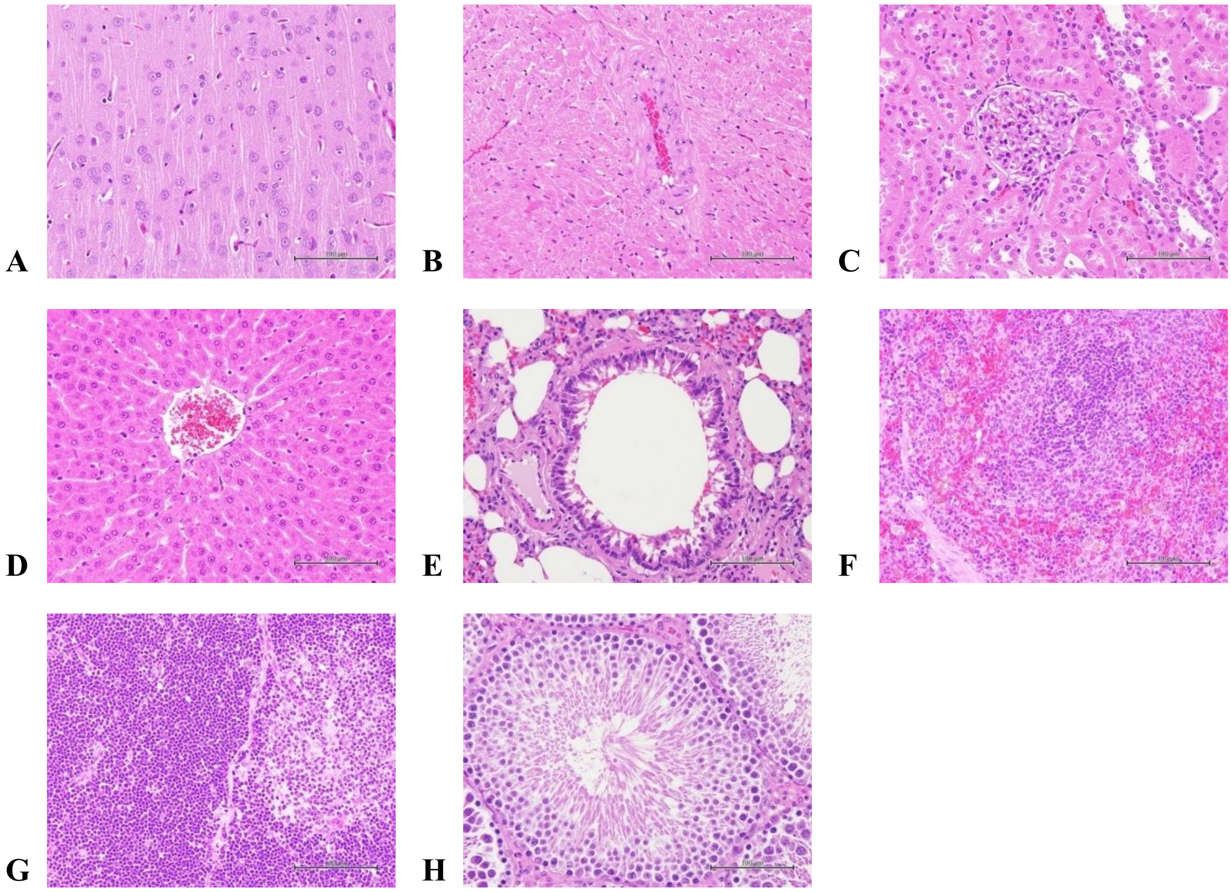

Figure S2. Histopathological appearances of the organs in high-dose ACP-treated rats. No significant lesions of brain (A), heart (B), kidney (C), liver (D), lung (E), spleen (F), thymus (G), and testis (H) (animal #137). H&E stain, 400x.

Table S1. Urinalysis findings of male rats receiving different doses of ACP for 90 days.

			ACP (mg/kg)		
		Control	200	600	1000
Male rats					
Appearance	Pale yellow	30[§]	30	20	40
	Yellow	70	60	80	60
	Orange	0	10	0	0
	Red	0	0	0	0
	Brown	0	0	0	0
Glucose	N	100	100	100	100
	P	0	0	0	0
Bilirubin	N	100	100	100	100
	1+	0	0	0	0
	2+	0	0	0	0
Ketone bodies	N	60	40	50	60
	+/-	40	60	50	40
	1+	0	0	0	0
	2+	0	0	0	0
Specific gravity	≤1.005	0	0	0	0
	1.005~1.030	50	30	40	50
	≥1.030	50	70	60	50
pH	≤5	0	0	0	0
	5~8	100	100	100	100
	≥8	0	0	0	0
Protein	N	0	0	10	0
	1+	70	50	60	70
	2+	30	50	30	30
	3+	0	0	0	0
Urobilinogen	N	100	100	100	100
	P	0	0	0	0
Nitrite	N	100	100	100	100
	P	0	0	0	0
Leukocytes	N	100	100	100	100
	P	0	0	0	0
Occult blood	N	100	100	100	100
	+/-	0	0	0	0
	1+	0	0	0	0
	2+	0	0	0	0

N: Negative; P: Positive; +/-: Trace

[§] Percentage (%): Number of rats observed/Total number of experimental rats x 100; n = 10.

Control: Received vehicle solution only.

Table S2. Urinalysis findings of female rats receiving different doses of ACP for 90 days.

		Control	ACP (mg/kg)		
			200	600	1000
Female rats					
Appearance	Pale yellow	30[§]	40	30	40
	Yellow	70	60	60	60
	Orange	0	0	10	0
	Red	0	0	0	0
	Brown	0	0	0	0
Glucose	N	100	100	100	100
	P	0	0	0	0
Bilirubin	N	100	100	100	100
	1+	0	0	0	0
	2+	0	0	0	0
Ketone bodies	N	100	70	80	100
	+/-	0	30	20	00
	1+	0	0	0	0
	2+	0	0	0	0
Specific gravity	≤1.005	0	0	0	0
	1.005~1.030	60	40	70	50
	≥1.030	40	60	30	50
pH	≤5	0	0	0	0
	5~8	100	100	100	100
	≥8	0	0	0	0
Protein	N	0	10	0	0
	1+	80	80	60	80
	2+	20	10	40	20
	3+	0	0	0	0
Urobilinogen	N	100	100	100	100
	P	0	0	0	0
Nitrite	N	100	100	100	100
	P	0	0	0	0
Leukocytes	N	100	100	100	100
	P	0	0	0	0
Occult blood	N	100	100	100	100
	+/-	0	0	0	0
	1+	0	0	0	0
	2+	0	0	0	0

N: Negative; P: Positive; +/-: Trace

[§]Percentage (%): Number of rats observed/Total number of experimental rats x 100; n = 10.

Control: Received vehicle solution only.

Table 3S. Summary of pathological incidence in organs of rats treated with or without ACP for 90 days.

		Control		High-dose ACP	
Organs	Histopathological findings	Male	Female	Male	Female
Adrenals	1.Infiltration, mononuclear cell, multifocal, slight[φ]	-	10[§]	-	-
	2.Hypertrophy, cholesterol, zona fasciculata, multifocal, slight to moderate/severe[1]	100	70	100	90
	3.Lipidosis, cytoplasmic, zona fasciculate, multifocal, slight	10	10	-	-
Heart	Infiltration, mononuclear cell, focal, minimal to slight[1]	20	-	10	-
Kidney		-	-	-	-
Liver		-	-	-	-
Lung	Collapse, alveolar, multifocal, slight to moderate/severe	100	100	100	100
Spleen		-	-	-	-
Testis		-	N	-	N
Ovary	Hypertrophy, cytoplasmic, interstitial gland, multifocal, slight to moderate	N	10	N	20
Thymus		-	-	-	-

High dose: 1000 mg/kg body weight; N: No tissue available; -. No effect.

[φ] Degree of lesions was graded from one to five depending on severity: 1 = minimal (< 1%); 2 = slight (1-25%); 3 = moderate (26-50%); 4 = moderate/severe (51-75%); 5 = severe/high (76-100%).

[§] Pathological incidence (%): Affected rats/Total number of experimental rats x 100; n = 10.

Control: Received vehicle solution only.
